# Transcriptomic and proteomic analyses reveal new insights into the regulation of immune pathways during adenovirus type 2 infection

**DOI:** 10.1186/s12866-018-1375-5

**Published:** 2019-01-14

**Authors:** Hongxing Zhao, Maoshan Chen, Alberto Valdés, Sara Bergström Lind, Ulf Pettersson

**Affiliations:** 1 The Beijer laboratory, Department of Immunology, Genetics and Pathology, Uppsala University, S-751 85 Uppsala, Sweden; 2 Australian Centre for Blood Diseases, Central Clinical school, Monash University, Clayton, Australia; 3 Department of Analytical Chemistry, Physical Chemistry and Chemical Engineering, University of Alcalá, Alcalá de Henares, Madrid, Spain; 4 Department of Chemistry-BMC, Analytical Chemistry, Uppsala University, Box 599, SE-751 24 Uppsala, Sweden

## Abstract

**Background:**

Human adenovirus (Ad) infection leads to the changes of host cell gene expression and biosynthetic processes. Transcriptomics in adenovirus type 2 (Ad2)-infected lung fibroblasts (IMR-90) cells has previously been studied using RNA sequencing. However, this study included only two time points (12 and 24 hpi) using constrained 76 bp long sequencing reads. Therefore, a more detailed study of transcription at different phases of infection using an up-graded sequencing technique is recalled. Furthermore, the correlation between transcription and protein expression needs to be addressed.

**Results:**

In total, 3556 unique cellular genes were identified as differentially expressed at the transcriptional level with more than 2-fold changes in Ad2-infected cells as compared to non-infected cells by using paired-end sequencing. Based on the kinetics of the gene expression changes at different times after infection, these RNAs fell into 20 clusters. Among them, cellular genes involved in immune response were highly up-regulated in the early phase before becoming down-regulated in the late phase. Comparison of differentially expressed genes at transcriptional and posttranscriptional levels revealed low correlation. Particularly genes involved in cellular immune pathways showed a negative correlation. Here, we highlight the genes which expose inconsistent expression profiles with an emphasis on key factors in cellular immune pathways including NFκB, JAK/STAT, caspases and MAVS. Different from their transcriptional profiles with up- and down-regulation in the early and late phase, respectively, these proteins were up-regulated in the early phase and were sustained in the late phase. A surprising finding was that the target genes of the sustained activators failed to show response.

**Conclusion:**

There were features common to genes which play important roles in cellular immune pathways. Their expression was stimulated at both RNA and protein levels during the early phase. In the late phase however, their transcription was suppressed while protein levels remained stable. These results indicate that Ad2 and the host cell use different strategies to regulate cellular immune pathways. A control mechanism at the post-translational level must thus exist which is under the control of Ad2.

**Electronic supplementary material:**

The online version of this article (10.1186/s12866-018-1375-5) contains supplementary material, which is available to authorized users.

## Introduction

Change of host cell gene expression and biosynthetic processes during a human adenovirus infection is a stepwise, but efficient mode of turning host antiviral responses to facilitate the replication of adenovirus. Most interactions between host cell and virus take place during the early phase. Adenovirus-mediated regulation of cellular gene expression emphasizes two major aspects: induction of its host cell to enter S-phase of the cell cycle and interference with host defense mechanisms. It has been shown that host cells are reprogrammed epigenetically as a result of adenovirus early-region function at different times after infection [1]. Adenovirus expresses several regulatory proteins from early regions 1A (E1A), E1B, E3, and E4. E1A is the first viral gene expressed and plays essential roles in regulation of viral and cellular gene expression [2]. E1A does not bind DNA directly by itself, but by binding to many important transcriptional regulators, E1A regulates the expression of important genes that control the cell cycle and cellular innate antiviral response [3–10]. The interaction of E1A with the retinoblastoma tumor suppressor (pRB) family proteins results in disassembling of a series of inhibitory complexes between pRB and the transcription factor E2F family, leading to the activation of E2F family of transcription factors. As consequence, the E2F-dependent S-phase genes are expressed [6, 7]. The interaction of E1A with the coactivators p300/CBP disrupts the histone acetyltransferase activity of p300/CBP and their associated factor PCAF, leading to decreased transcription from a variety of different genes involved in growth arrest, cell differentiation and immune evasion [4, 5, 8, 9, 11–14]. Furthermore, E1A proteins interfere with host immune response by blocking type I IFN-inducible gene expression [15]. E1A protein directly antagonizes a cellular histone posttranslational modification mediated by hBre1/RNF20, thus inactive the cellular IFN-stimulated gene (ISG) expression [16]. E1A associates with hypophosphorylated pRB1 and p300/CBP and translocate the complex to the gene bodies of repressed genes [17]. Many components of TGFβ-, TNF-, and interleukin-signaling pathways are among their targets. A recent study shows that the E1A C terminus interacts with three cellular proteins FOXK, DCAF7 and CtBP and suppress activation of a subset of ISGs [18]. In addition, it has been shown that E1A protein prevents the peptide presentation to the immunoproteosome by interacting with MECL1 [19].

E1B encodes two major proteins, the E1B-55 K and E1B-19 K proteins. E1B-55 K is a multi-functional protein and plays a major role in counteracting the cellular proapoptotic program. Association of E1B-55 k and E4-orf6 proteins with several cellular proteins, Cullin 5, TCEBs and RBX1 forms a virus-specific E3 ubiquitin ligase which then targets specific cellular proteins for degradation [20, 21]. The E1B-55 K protein serves as the substrate-recognition subunit via distinct sequences and targets the p53 protein, thereby promoting degradation of p53 [21, 22]. The E1B-19 K protein, a viral Bcl-2 homologue, interferes directly with the activity of p53 when translocated into the mitochondria [23–25]. Proteins generated from the E3 region also play a very important role in countering host antiviral defenses [26]. E3-gp19K prevents the exposure of viral peptides on the cell surface by blocking the transport of the class I major histocompatibility complex (MHC I) molecule to the cell surface and the loading of peptides by tapasin [27–29]. The E3-10.4 K and 14.5 K (RIDα/β) complex inhibits tumor necrosis factor alpha (TNFα) and Fas ligand-induced apoptosis through internalization and degradation of the death domain containing receptors [30]. In addition, the E3-10.4 K/14.5 K complex blocks the activation of NFκB by preventing it from entering the nucleus and inhibiting the activity of the kinase complex IKK [31]. Proteins encoded by the E4 region are involved in transcriptional regulation. E4-orf6/7 stabilizes the binding of E2F to the duplicated E2F binding sites in the E2 promoter [32, 33]. E4-orf3 associates with E1B-55 K in the nuclear promyelocytic leukemia protein oncogenic domains (POD) and reorganizes PODs during infection, thus likely involved in the regulation of transcription factor availability and activity [34, 35]. The E4-orf4 protein interacts with protein phosphatase 2A, leading to the inhibition of E1A-dependent transactivation of the JunB promoter [36–38].

When adenovirus DNA replication commences, the infection cycle proceeds into the late phase. Viral transcription changes from the early to the late pattern. The L4-100 kDa protein, expressed from the major late transcription unit is necessary for efficient initiation of viral late mRNA translation [39–41]. Furthermore, the E1B-55 kDa and E4-orf4 protein complex is involved in regulation of mRNA export from nucleus, resulting in a block of cellular mRNAs export and selective export of viral mRNAs [42, 43]. As a consequence, a dramatic down-regulation of cellular gene expression occurs late in infection [44].

Most studies of the adenovirus infection have been performed in Hela cells, in which adenovirus replication is very efficient and the infectious cycle is completed after 20–24 h [45]. Particularly, the early phase is very short, lasting for less than 6 h. Thus, there is a narrow time window for a detailed examination of the changes of cellular gene expression. Furthermore, being transformed cells, Hela cells grow rapidly and are difficult to synchronize. Thus, genes involved in the control of cell cycle and growth might escape detection. Therefore, human primary cells, like human lung fibroblasts (IMR-90) or foreskin cells (HFFs) have been used for a series of studies [44, 46–49]. In these cells adenovirus DNA replication starts 24 h post infection (hpi). Based on cellular transcription profiles from early cDNA microarray study, Ad2 infection of IMR-90 cells can be divided into four periods [44]. The first period (1–12 hpi) extends from the attachment of Ad2 to the cell surface to the beginning of adenoviral early gene expression. During this time, the cellular gene expression changes are mainly triggered by the virus entry process. The majority of the genes deregulated during the first phase have functions linked to cell growth and immune response. The second period covers the time from the expression of the immediate early E1A gene to the time when Ad2 DNA replication starts (12–24 hpi). During this period, there is a linear increase in the number of differentially expressed cellular genes involved primarily in cell cycle regulation and cell proliferation. The third period ranges from the beginning of DNA replication to the time when the cytopathic effect (CPE) starts (24–36 hpi). By this time, the virus has gained control of the cellular metabolic machinery, resulting in an efficient replication of the viral genome and expression of the capsid proteins. Additional changes in cellular gene expression are modest during this phase. The final period starts when CPE is apparent (after 36 hpi). The number of down-regulated genes increases dramatically and includes many genes involved in intra- and extracellular structure, leading to an efficient burst of progeny.

The rapid development of high throughput sequencing technology enabled the exploration of the transcriptome on a genome-wide scale at single base pair resolution. Meanwhile, several proteomics approaches have been applied. Improve shotgun/bottom-up liquid chromatography-tandem mass spectrometry (LC-MS/MS)-based protein detection and quantitative techniques such as Stable Isotope Labelling of Amino acids in Cell culture (SILAC) have greatly facilitated protein identification [50, 51]. These technologies have been used in studies of protein expression in adenovirus-infected cells. Lam et al. have analyzed the nucleolar proteome in Ad5-infected Hela cells [52], while Evans et al. have examined the posttranscriptional stability of cellular protein in Ad5-infected Hela cells [53]. Recently, a comparative proteome analysis of wild type and E1B-55 K-deleted viruses was performed to investigate the role of Ad5 E1B-55 K in targeting cellular proteins with antiviral activity for proteasomal degradation [54]. Previously, we have presented a general comparison of the cellular transcriptome and proteome of Ad2-infected IMR-90 cell at 24 and 36 hpi [48]. More than 700 proteins were identified to be differentially expressed. Surprisingly, there was a very low correlation between the RNA and protein expression profiles. Here, we present a more comprehensive study of the cellular transcription profiles at four critical stages of an adenovirus infection in IMR-90 cells using paired-end sequencing. As a step further, RNA expression profiles were compared with protein expression profiles with a focus on genes involved in the cellular immune response.

## Materials and methods

### Cell culture and virus infection

Human lung fibroblast IMR-90 cells (American Type Culture Collection, ATCC) were initially cultured in Eagle’s minimum essential medium (EMEM) (ATCC) supplemented with 10% fetal bovine serum (FCS), 100 U/ml penicillin and 100 μg/ml streptomycin at 37 °C and 5% CO_2_. After reaching conluent, cells were maintained in the plates for two days before infection. By fluorescence-activated cell sorting (FACS) analysis, more than 95% of the cells were characterized in G0/G1 phase. Synchronized cells were then infected with human adenovirus type 2 at a multiplicity of infection (MOI) of 10. Mock-infected cells were used as a control. One hour later, the medium was replaced with complete EMEM medium supplemented with 10% FBS. Infected cells were collected at 6, 12, 24, and 36 h post infection (hpi).

### Total RNA extraction, RNA library construction and sequencing

Total RNA from infected IMR-90 cells were extracted with TRIzol® (Invitrogen), according to the manufacturer’s instructions. The quality of total RNA was evaluated with a NanoDrop 1000 spectrophotometer and an Agilent 2100 Bioanalyzer. After treatment with Ribo-Zero™ rRNA removal reagent, total RNA was used to construct cDNA library for transcriptome sequencing following the ScriptSeq™ v2 RNA-Seq library preparation kit according to the manufacturer’s protocol (Epicentre). The cDNA libraries were sequenced on a HiSeq 2000 sequencing platform (Illumina).

### Genome alignment and gene expression profile

Data cleaning was performed by removing low quality, contaminant and adapter reads from the raw reads. TopHat2 and Cufflinks were used to align the filtered reads to human Ensembl genome (http://www.ensembl.org/index.html, GRCh38) and to profile gene expression following the protocol [55], respectively. FPKM (fragments per kilobase per million reads mapped) method was employed to normalize gene expression. To strengthen the reliability of our results, lowly expressed genes (< 10 FPKM in all libraries) were filtered out.

### Identification of differentially expressed genes in Ad2-infected cells

To identify genes deregulated in early and late phases of Ad2 infection, we performed correlation analysis between samples based on normalized gene expression values using the CORREL function provided by Excel. To identify differentially expressed genes in the cells infected by Ad2, several statistical values were used. First, a fold change of a particular gene in Ad2-infected cells was calculated following the rule: fold change (Ad2-infected/mock) = y/x, while y and x represent the normalized expression values in Ad2-infected and mock cells, respectively. A cut-off of more than 2-fold increase or decrease was used. Second, a *p*-value that represents the significance for differential expression was calculated based on Poison distribution [56]. A cut-off for *p*-values (< 0.05) was used for differentially expressed genes. Last, an R package called NOISeq was used to calculate the probability of differential expression of a gene in a comparison [57]. Only those genes with probability > 0.7 were kept for further analysis.

### Gene ontology and KEGG pathway enrichment

To determine the biological processes and KEGG pathways affected by human adenovirus type 2, differentially expressed genes were analyzed by DAVID Bioinformatics Resources 6.7 (http://david.abcc.ncifcrf.gov/) [58].

### SILAC-MS experiment and protein identification

The protein labelling were performed as described before [48]. Briefly, after growing in cell culture medium containing with heavy or light amino acids for at least six passages, cells were mock infected or infected with Ad2 at MOI of 10 in serum-free medium [59]. A biological replicate with swapped labeling was also performed. After harvest, cells were lysed and mock- and Ad2-infected lysates of different labeling were combined in a 1:1 protein ratio. Proteins were fractionated using SDS-PAGE. Following in-gel tryptic digestion [60], peptides were extracted and analyzed using nano liquid chromatography coupled on-line to a QExactive Orbitrap Plus Mass spectrometer (ThermoFisher Scientific, Bremen,Germany). Acquired data (raw-files) were imported into MaxQuant software (version:1.4.5.7) [61], and searched against a FASTA-file containing both cellular and Ad2 proteins downloaded from UniProt 2017–02. The ratio of the chromatographic areas of heavy and light peptides matching to specific proteins was used for determining the protein expression levels.

## Results

### Host cell transcriptional profiles during the course of an adenovirus infection

Regulation of cellular transcription during Ad2 infection was studied using paired-end sequencing technology. Four infection time points, 6, 12, 24 and 36 h post infection (hpi), were chosen which represent different stages of Ad2 infection. Besides, all of our early studies on expression of cellular various RNAs including micro RNA (miRNA), long non-coding (lncRNA) and protein were performed under the same condition [44, 46, 47]. Thus, we could compare the various expression profiles. About 30 million 255 bp long sequence reads per sample were generated and 53–58% of them accounted for mRNA. From them 6860 cellular genes were identified to be transcribed at a significant level with a minimum of 10 FPKM (fragments per kilobase per million reads mapped) (Table 1). Among them, expressions of 3556 genes were changed more than or equal to 2-fold with *p*-values< 0.05 in infected cells as compared to non-infected cells. Very limited changes in RNA expression occurs during the early phases. Only 74 and 223 genes showed significant differential expression at 6 and 12 hpi, respectively. Most expression changes took place at 24 hpi when infection proceeded into the late phase, 2239 and 3060 genes were differentially expressed at 24 and 36 hpi, respectively. Fewer differentially expressed genes were detected in this study as compared to our earlier study, in which 1267 and 3683 cellular genes were identified as differentially expressed at 12 and 24 hpi. However, the former study was less stringent and included genes covered with only one or more reads [62].

**Table 1:** In total 12,927 cellular mRNAs were detected in five time points together. Among them 9738 mRNAs were common between all-time points. Expression of 6860 mRNAs reached to a significant level with a minimum of 10 FPKM. Among them 3556 mRNAs were up- or down-regulated ≥2-fold in Ad2-infected cells as compared to non-infected cells. Numbers of mRNAs at each time point are listed here

Selection	Mock	Ad2–6 hpi	Ad2–12 hpi	Ad2–24 hpi	Ad2–36 hpi
≥ 1 FPKM	11,064	11,163	11,837	11,426	11,191
≥ 10 FPKM	5001	4846	5184	4692	4371
≥ 2-Fold change		74^a^	223	2239	3060
Up-regulated		65^b^	138	1694	2142
Down-regulated		9^c^	85	545	918

^a^Number of genes expression with more than 2-fold changes in Ad2-infected as compared to uninfected cells as measured by sequence reads. ^b^Number of more than 2-fold up-regulated mRNAs in Ad2-infected as compared to non-infected cells. ^c^Number of more than 2-fold down-regulated mRNAs in Ad2-infected as compared to non-infected cells

**Fig. 1:**
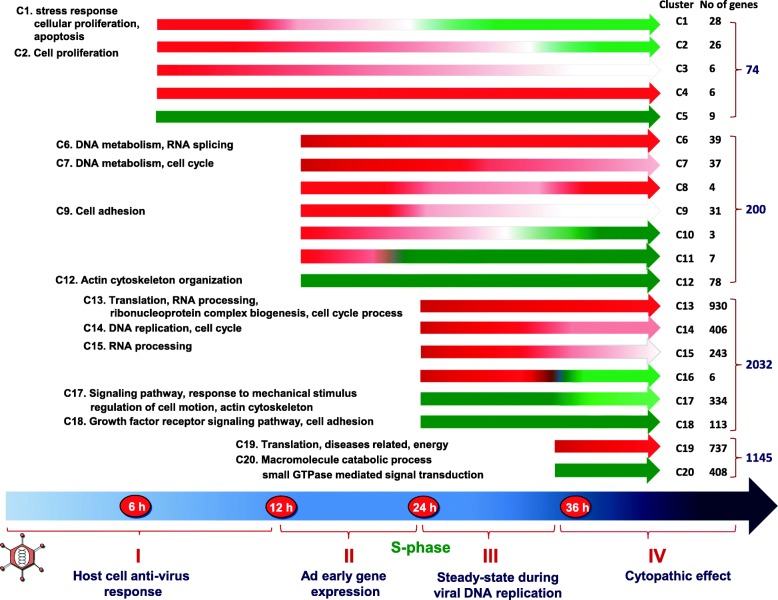
Based on the kinetics of transcription changes, the differentially expressed genes were grouped into 20 clusters (C1-C20). The numbers of differentially expressed genes identified at each time point and in each cluster were indicated on the right hand side (Note that the numbers of genes at 12, 24 and 36 hpi are different to the Table 1 because many genes were identified as differentially expressed at more than one time point, but included only in one cluster). The biological functions of genes in each cluster were analyzed using DAVID (left). Red, green and white arrow bars represent RNAs that were up-, down-regulated or unchanged in Ad2-infected cells in comparison to RNA in uninfected control

Based on the kinetics of change in gene expression at different stages of infection, 3451 out of 3556 genes fell into 20 major different expression clusters (Fig. 1). The complete list of genes in each cluster is included in Additional file 1: Table S1. At 6 hpi, more than 87% of the differentially expressed genes were up-regulated (Clusters 1 + 2 + 3 + 4). Expression of all of these genes reached their highest levels at 6 hpi, except two which reached their highest levels at 12 hpi. Then about 80% of them became down-regulated during the late phase of infection (Clusters 1 and Cluster 2). The rest either remained up-regulated (Cluster 4), or were gradually reduced to the basal level in the late phase (Cluster 3). Only 9 genes (Cluster 5) were down-regulated at 6 hpi and their expression remained suppressed until the late phase.

At 12 hpi, 122 and 78 genes became up- and down-regulated in addition to the differentially expressed genes since 6 hpi. Among the up-regulated genes, about 1/3 increased until 36 hpi (Cluster 6), 1/3 remained at a similar level through the rest of the infection (Cluster 7), and the remaining 1/3 was only transiently up-regulated at 12 hpi (Cluster 9) and became down-regulated at 24 hpi (Cluster 11) or at 36 hpi (Cluster 10). Except one gene, all down-regulated genes at 12 hpi remained suppressed until the late phase (Cluster 12).

The most dramatic changes in gene expression took place between 12 to 24 hpi then the infection proceeded from the early to the late phase. Thus, expression of 1585 and 447 (2032 in total) additional genes was up- and down-regulated at 24 hpi. Based on the expression changes at 36 hpi, the up-regulated genes at 24 hpi fell into four profiles (Cluster 13 + 14 + 15 + 16). Expression of 59% of these genes increased until 36 hpi (Cluster 13), whereas 25% decreased but remained > 2-fold higher than in non-infected cells (Cluster 14) and 15% declined to less than 2-fold changes at 36 hpi (Cluster 15). Only 6 genes became down-regulated at 36 hpi (Cluster 16). Among 447 down-regulated genes, 75% decreased continually until 36 hpi (Cluster 17), while 25% remained at a similar level (Cluster 18). Change in cellular gene expression was modest between 24 to 36 hpi as compared to that between 12 to 24 hpi. In comparison to non-infected cells, expression of 737 (Cluster 19) and 408 genes (Cluster 20) became up- or down-regulated at 36 hpi in addition to the genes that had been differentially expressed since 12 or 24 hpi.

### Biological functions of genes in different expression clusters

The biological consequences of the gene expression changes were analyzed using DAVID (The Database for Annotation, Visualization and Integrated Discovery) and are shown in Fig. 1 (left hand panel), and more detailed results are included in Additional file 1: Table S1. No significant functional categories can be identified by DAVID if the cluster contained few than 20 genes. The most significant functions of the genes in clusters 1 and 2 were stress response, cellular proliferation and apoptosis. A significant group of genes were cytokines, such as CXCL1, IL6, CCL2, CCL20, CXCL3, TNFSF15, IL1B, HGF, IL11, CXCL10, RALA, FGF2, FGF7, GDF15, AREG and HGF involving stress/immune response and cell growth control. Several genes that involved in apoptosis included MDM2, TNFAIP2, TNFAIP3, TNFAIP6, TNFAIP8, TNFSF15 and BIRC3 and involved in cell cycle control, such as CDKN1A, FYN, PLK2, AHR, and RGCC, were also noteworthy. Among transcription factors, up-regulation of ATF3 was the most significant and it increased 6-fold compared to the non-infected control. Expression of ATF3 has been shown to be induced by a variety of signals and it is involved in cellular stress response. Only 9 genes were present in cluster 5 and therefore no significant functional categories could be identified by DAVID. However, four (PTPN12, MAP4K3, ERRFI1 and LBH) out of the 9 genes, are involved in cellular signaling and growth control.

During the period between 6 and 12 hpi, adenovirus early genes begin to be expressed, redirecting cellular gene expression. The up-regulated cellular genes are involved in DNA replication (Clusters 6 and 7), including Minichromosome Maintenance Complex Components (MCM) 3, 4, 5, 6, 7 and components of the post-replicative DNA mismatch repair system (MMR) alpha (MSH2-MSH6 heterodimer). In addition, genes implicate in transcription and pre-RNA processing were prominent in cluster 6. Genes implicated in cell cycle were significant in Cluster 7, including CDC25A, CCNE2, CCNE1 and CDK2, the key regulators for the progression from G1 to the S phase. Although no significant function was identified for clusters 8 to 11, several genes, such as JunB, GADD45B and PAPPA function in control of cell growth and proliferation were included in this cluster. The most significant function for the down-regulated genes was actin cytoskeleton organization.

There was a dramatic increase in the number of differentially expressed genes between 12 and 24 hpi. Cellular genes which function in protein translation became significant among up-regulated genes. These genes covered both cytoplasmic and mitochondrial ribosomal proteins, eukaryotic translation initiation and elongation factors. Although genes involved in DNA replication and cell cycle were still significant, similar to those at 12 hpi, the number of genes in these categories increased dramatically. For instance, the number of genes involved in DNA metabolism/DNA replication increased from 18 to 124, whereas genes implicated in cell cycle increased from 19 to 153. Most of these genes present in clusters 13, 14 and 15. The large number of genes involved in the cell cycle included many key regulators, such as E2Fs, cyclins, cyclin dependent kinases and cell division cycle (CDC) genes. In addition, genes participating in RNA processing became significant. Several important components of the exosome complex involved in the degradation and processing of a wide variety of RNA species were also up-regulated.

The number of down-regulated genes between 12 and 24 hpi also increased (Clusters 17 and 18). The most significant function of genes in these clusters was various growth factors and receptors. Cytoskeleton organization was significant for genes in cluster 17, whereas genes implicated in cell adhesion were significant in cluster 18. Previous experiments have shown that the replication of Ad2 DNA reaches a maximum rate during the period from 24 to 36 hpi [44]. However, cellular gene expression was still maintained at a high level. The most significant function of the up-regulated genes (Cluster 19) was protein translation similar to that at 24 hpi, but with an increased number of genes. Genes involved in the generation of precursor metabolites and energy, as well as oxidation reduction became significant. In addition, several genes identified in different diseases were also significant. The major function for the down-regulated genes (Cluster 20) was cellular macromolecule catabolic processes such as ubiquitination and subsequent proteasome degradation of target proteins. Another significant function was small GTPase mediated signal transduction, involved in vesicle transport.

### Consensus transcription factor binding sites in the promoter region of genes in the different clusters

Genes sharing a similar transcription profile are likely to be regulated by common transcription factors (TF) or TFs from the same family. To this end, the genes in the 20 different clusters were subjected to analysis for the presence of consensus TF binding sites in their promoter regions (− 300 to + 100) using Transfind [63]. The most over-represented TF binding sites are listed in the order of significance in Table 2. NFκB and c-Rel binding sites were most significant for the genes in cluster 1. Interesting genes among them were BIRC3, IKBA, CCL20, GROA (CXCL1), TNAP3 and TNF15, known to be involved in immune response or apoptosis. No significant enrichment of TF binding sites was identified for the genes in clusters 2, 3, 4, 5. For the genes in clusters 6 and 7, only the E2F binding site was significant. More genes with E2F binding were identified in clusters 13 and 14. In addition, the binding sites for GABP, NRF1 and ATF/CREB family were significant among genes in clusters 13, 14 and 15, respectively. GABP regulates genes that are involved in cell cycle control, protein synthesis, and cellular metabolism. NRF1 activates the expression of key metabolic genes regulating cellular growth. The ATF/CREB family has diverse functions in controlling cell proliferation and apoptosis. In contrast, the TF binding sites among the down-regulated genes were less significant. Only the MZF1 and AP2 binding sites were scored but their significance was low and they were only present on 8 or 7 genes, respectively. MZF1 can function as a tumor/growth suppressor and controls cell proliferation and tumorigenesis [64]. At 36 hpi, different sets of TF binding sites became significant for up-regulated genes (Cluster 19), including SP1, STRA13 and NF-Y in addition to GABP while the binding sites for E2F became less significant. This correlated very well with the expression profile of E2Fs. Expression of all E2Fs increased at 12 and 24 hpi, and then decreased at 36 hpi. The TF binding sites for the down-regulated genes were less significant and STRA13 and USF were on the top of the list. STRA13 is a transcriptional repressor. Correspondingly, its expression increased 4 and 8 times at 24 and 36 hpi, respectively. STRA13 is involved in DNA damage repair and genome maintenance. Surprisingly, the STRA13 binding site was significant for both up- and down-regulated genes at 36 hpi. Its transcriptional repression is probably mediated by recruitment of other regulatory factors and, depending on the cofactors, STRA13 plays divergent roles. USF that binds to a symmetrical DNA sequence (E-boxes; 5-CACGTG-3) is involved in the transcriptional activation of various genes implicated in physiological processes, such as stress response, immune response, cell cycle control and tumor growth.

**Table 2:** Presence of consensus transcription factor binding sites in the − 300 to + 100 promoter sequence of differentially expressed genes in different clusters

	Rank	Binding site	FDR	No. genes^a^
Cluster 1	1	NF-kappaB	0.000094	6
	2	c-Rel	0.001353	5
Cluster 6	1	E2F	0.005309	5
Cluster 7	1	E2F	0.000527	6
Cluster 13	1	E2F	< 0.000001	99
	2	GABP	< 0.000001	49
	3	NRF1	< 0.000001	40
	4	CREBP1CJUN/ATF2:c-Jun	< 0.000001	35
	5	AHRHIF/AhR,	< 0.000001	35
	6	STAT1	< 0.000001	34
	7	CREBATF	< 0.000001	33
Cluster 14	1	E2F	< 0.000001	42
	2	CREBP1CJUN/ATF2:c-Jun	< 0.000001	26
	3	NRF1/NRF-1	< 0.000001	24
	4	CREB	< 0.000001	24
	5	ATF3	< 0.000001	22
	6	CREBATF	< 0.000001	22
	7	CREBP1/ATF2	< 0.000001	20
	8	ATF1	< 0.000001	20
Cluster 15	1	CREB	0.000004	15
	2	HIF1	0.000007	14
	3	ATF3	0.000007	14
	4	CREBATF	0.000007	14
	5	E2F	0.000037	25
	6	CREBP1/ATF2	0.000159	12
	7	STAT1	0.000559	11
	8	ATF1	0.000559	11
Cluster 17	1	CREBP1/ATF2	0.000257	16
	2	CREB	0.007176	13
	3	CREBATF/CREB,	0.007176	13
	4	SP1	0.013579	12
	5	TFII-I	0.013579	12
	6	AHRHIF/AhR,	0.013579	12
	7	CREBP1CJUN/ATF2:c-Jun	0.02189	11
	8	EGR1/Egr-1	0.02189	11
Cluster 18	1	MZF1	0.00585	8
	2	AP-2	0.021453	7
Cluster 19	1	SP1	< 0.000001	33
	2	GABP	< 0.000001	28
	3	STRA13	0.000023	25
	4	NF-Y	0.000053	24
	5	NRF-1	0.000053	24
	6	E2F	0.000132	31
	7	USF	0.000334	34
	8	CREBATF/CREB,	0.000334	22
	9	NFY	0.000845	21
	10	Egr-1	0.002093	20
Cluster 20	1	Stra13	< 0.000001	23
	2	USF	0.000007	19
	3	c-Myc:Max	0.000024	18
	4	CREBP1CJUN/ATF2:c-Jun	0.000304	16
	5	CREBATF/CREB,	0.000304	16
	6	GABP	0.00081	15
	7	ATF6	0.00081	15
	8	ATF4	0.00081	15
	9	ATF1	0.00273	14

^a^The number of genes contain the consensus TR binding site

### Comparison of RNA and protein expression profiles for the genes involved in cellular immune pathways

**Fig. 2:**
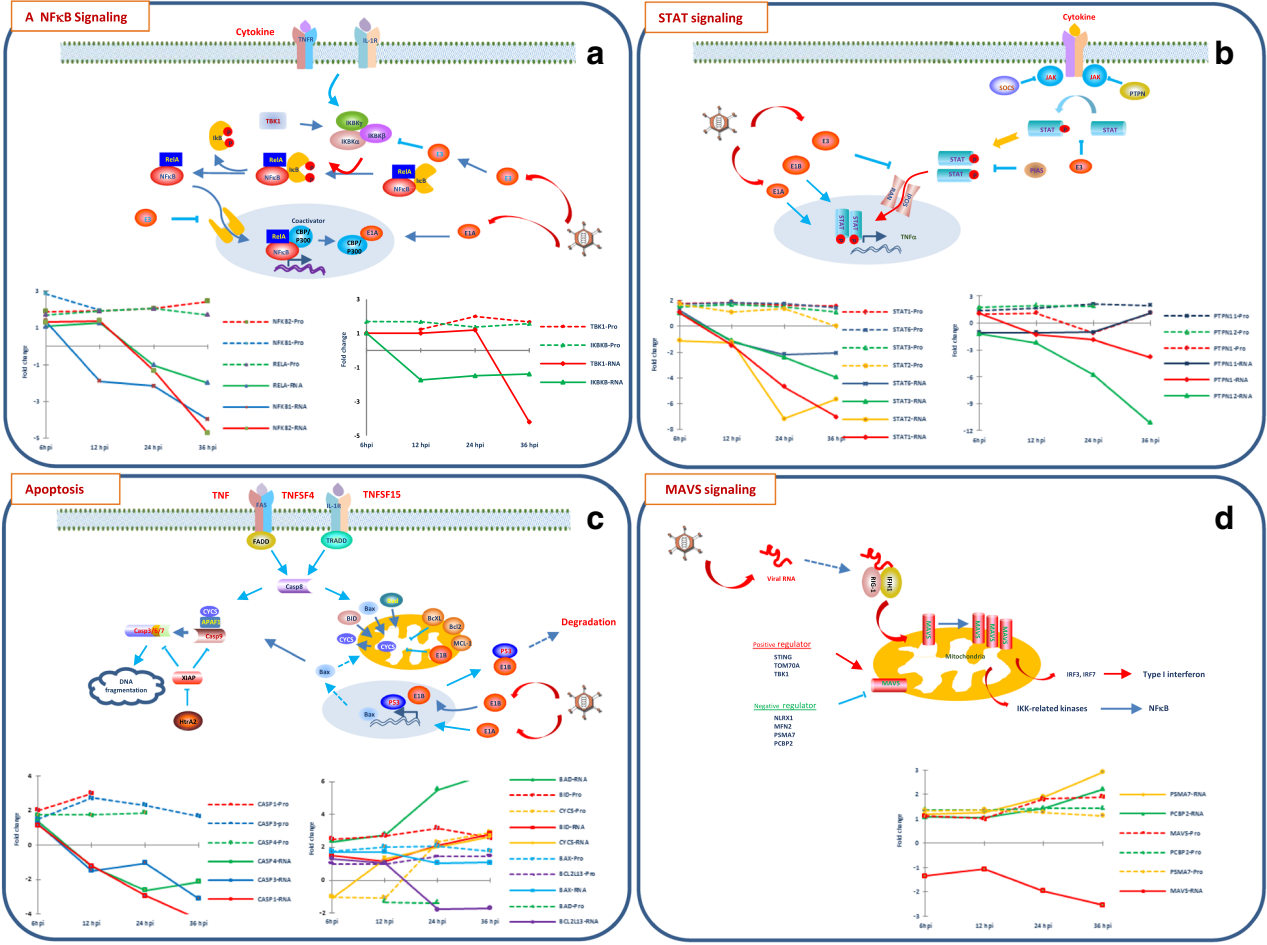
Schematic representation of the expression profiles of key components of NFκB (**a**), STAT (**b**), apoptosis (**c**) and MAVS (**d**) pathways. The involvement of Ad2 proteins is also indicated. Graphs display the expression profiles (fold change between Ad2 and mock) of genes that detected at both protein (dash line) and RNA (solid line) levels at 4 time points. The same color is used for the corresponding protein and RNA. Most of these proteins were up-regulated at 6 hpi and sustained until late phase, while their RNA level increased at 6 hpi and then decreased at 12 or 24 hpi until late phase

Cellular immune network is a major target during Ad2 infection. As shown above, the transcription of genes involved in immune response displayed very dramatic changes, being transiently up-regulated at 6 hpi before becoming down-regulated after 24 hpi. The expression of these genes at protein level was also analyzed. The protein expression data was retrieved from our early study [48, 65]. Our previous data analysis of the functions of differentially expressed proteins was mainly relied on a web-based tool DAVID, a functional enrichment analysis by integrating wide-range heterogeneous data. Thus, it is less ideal for analysis of virus-induced changes in gene expression because of underrepresentation of genes related to virus infection. Specifically, the expression of proteins involved in cellular immune pathways remained to be studied. Here, we identified many key regulators in cellular immune pathways that displayed inconsistent expression profiles between RNA and protein expression as listed in Table 3. Their expression profiles are shown in Fig. 2.

**Table 3:** Expressions of genes involved in cellular immune pathways at the RNA and protein levels

Pathway	Entrez GeneID	Gene symbol	RNA expression									Protein expression Fold change							
			Seq Reads (RPKM)					Fold change											
			Mock	Ad2–6 h	Ad2–12 h	Ad2–24 h	Ad2–36 h	Ad2–6 h/M	Ad2–12 h/M	Ad2–24 h/M	Ad2–36 h/M	Ad2–6 h/M	Ad2–6 h/M^a^	Ad2-12 h/M	Ad2-12 h/M	Ad2–24 h/M	Ad2–24 h/M	Ad2–36 h/M	Ad2–36 h/M
NFκB pathway	5966	REL	3,11	1,29	4,44	6,78	3,25	-2,41	1,43	2,18	1,04								
	5970	RELA	8,34	9,07	10,56	8,2	4,18	1,09	1,27	-1,02	-1,99	1,49	1,94	1,94	1,89	1,66	2,46	1,83	1,59
	5971	RELB	2,57	4,96	3,58	2,21	0,24	1,93	1,39	-1,17	−10,89								
	4790	NFKB1	23,98	32,77	12,61	11,09	6,03	1,37	−1,9	−2,16	−3,97		2,86	2,49	1,43				
	4791	NFKB2	7,76	10,13	10,81	5,85	1,65	1,31	1,39	−1,33	−4,71	1,88		1,96	1,85	2,13	1,97	2,44	
	4792	NFKBIA	27,49	50,03	39,55	10,73	2,58	1,82	1,44	−2,56	−10,66								
	4793	NFKBIB	2,96	3,5	5,4	23,03	50,04	1,18	1,83	7,79	16,93								
	4794	NFKBIE	3,02	2,43	1,7	0,48	0,42	−1,24	−1,77	−6,31	−7,19								
	64,332	NFKBIZ	12,38	24,41	6,08	5,16	1,38	1,97	−2,04	−2,4	−8,97								
	3551	IKBKB	9,11	9,27	5,28	6,14	6,55	1,02	−1,73	−1,48	−1,39	1,71	1,68	1,75	1,6	1,37		1,72	1,41
	9641	IKBKE	3,86	4,55	2,2	0,21	0,16	1,18	−1,76	−18,14	− 24,8								
	8517	IKBKG	8,83	8,22	7,72	3,31	2,86	−1,07	−1,14	−2,67	−3,09								
	29,110	TBK1	15,46	15,62	14,59	18,34	3,68	1,01	−1,06	1,19	−4,2			1,37	−1,09	2,48	1,49	2,01	1,32
	121,457	IKBIP	62,38	64,66	86,35	67,76	117,18	1,04	1,38	1,09	1,88	−1,1	− 1,08	−1,16	− 1,12	− 1,12	−1,17	− 1,31	
	8518	IKBKAP	17,63	12,99	24,1	28,2	24,4	−1,36	1,36	1,6	1,38	1,45			1,47	1,23	2,04	2,69	
STAT pathway	6772	STAT1	47,15	49,36	31,95	10,04	6,71	1,05	−1,48	−4,7	−7,02	1,56	2,03	1,8	1,89	1,66	1,66	1,79	1,32
	6773	STAT2	12,37	11,16	9,95	1,73	2,19	−1,11	−1,24	−7,15	−5,64	1,87	1,57	2,2			1,37		
	6774	STAT3	20,7	21,27	18,48	8,62	5,23	1,03	−1,12	−2,4	−3,95	1,54	1,49	1,79	1,62	1,34	1,64	1,2	−1,04
	6776	STAT5A	1,05	1,53	1,31	0,69	0,38	1,46	1,24	−1,53	− 2,78								
	6777	STAT5B	8,18	6,84	6,43	3,92	2,94	−1,2	−1,27	−2,08	−2,78								
	6778	STAT6	15,13	18,39	12,39	6,96	7,24	1,22	−1,22	−2,17	−2,09	1,44	1,93	1,87	1,82	1,57	1,91	1,44	
	10,379	IRF9	12,66	11,52	9,55	2,55	1,76	−1,1	−1,33	−4,97	−7,21								
	11,099	JAK1	39,584	57,628	39	30,45	36,29	1,46	−1,02	−1,3	− 1,09	−1,11	− 1,09	−1,43	− 1,24	1,65			
	3717	JAK2	12,928	9601	20,03	2,39	2,67	−1,35	1,55	−5,42	−4,84								
	7297	TYK2	4606	3584	7,04	6,44	8,41	−1,29	1,53	1,4	1,83								
	79,711	IPO4	8,37	11,34	14,38	18,45	27,93	1,35	1,72	2,2	3,33	1,3	1,94	1,83	1,91	2,21	2,71	3,11	2,61
	3843	IPO5	70,6	64,12	77,09	140,1	219,49	−1,1	1,09	1,98	3,11								
	10,527	IPO7	81,4	76,2	68,65	88,61	56,74	−1,07	− 1,19	1,09	− 1,43	1,51	1,73	1,7	1,62	1,92	2,26	1,99	2,14
	10,526	IPO8	11,77	8,17	11,68	6,02	2,99	−1,44	− 1,01	− 1,96	−3,94	1,39	1,38		1,28	1,35	1,51		1,01
	55,705	IPO9	8,29	7,62	9,23	10,92	11,82	−1,09	1,11	1,32	1,43	1,81	2,11	2,14	1,92	1,62	2,09	1,77	1,44
	5901	RAN	218,35	243,71	271,63	610,2	752,62	1,12	1,24	2,79	3,45	1,56	1,86	2,13	1,88	2,12	2,09	2,03	1,84
	5770	PTPN1	29,58	31,19	23	15,66	7,69	1,05	− 1,29	−1,89	−3,85	1,05	1,01	1,11	−1,08	− 1,08	− 1,2	1,06	− 1,3
	5771	PTPN2	11,56	14,23	14,25	8,87	5,48	1,23	1,23	−1,3	−2,11								
	5780	PTPN9	16,03	15,26	15,05	12,07	5,35	−1,05	− 1,07	− 1,33	−3								
	5781	PTPN11	107,55	101,5	98,71	103,4	121,24	−1,06	− 1,09	− 1,04	1,13	−1,04	1,82	1,66	1,68	1,91	2,32	2,27	1,68
	5782	PTPN12	28,93	24,12	13,15	5	2,6	−1,2	−2,2	−5,79	− 11,14	1,74		1,71	2,18	1,54	2,24		
	5783	PTPN13	9,93	8,53	12,35	5,85	4,33	−1,16	1,24	−1,7	−2,29								
	5784	PTPN14	28,65	20,22	11,72	3,63	2,14	−1,42	−2,45	−7,88	− 13,39								
	26,469	PTPN21	12,43	11,43	5,8	1,73	0,96	−1,09	−2,14	−7,18	− 12,94								
	6714	SRC	1,64	1,82	1,77	3,04	0,72	1,11	1,08	1,85	−2,78					−1,14	1,11	1,10	−1,22
	9636	ISG15	3,6	5,0	6,5	13,9	25,8	1,4	1,8	3,8	7,1								
	2537	IFI6	8,2	10,5	17,1	14,1	43,6	1,3	2,2	1,7	5,3								
Apoptosis	9966	TNFSF15	3,25	22,25	1,23	0,13	0,36	6,85	−2,65	−25,14	−8,91								
	7292	TNFSF4	13,65	11,23	2,78	0,71	–	−1,22	−4,92	−19,28	− 13,65	− 1,12	− 1,18						
	4982	TNFRSF11B	264,00	338,58	100,00	10,67	10,15	1,28	−2,64	−24,75	−26,02								
	51,330	TNFRSF12A	154,04	189,38	108,11	23,96	25,90	1,23	−1,42	−6,43	−5,95								
	8793	TNFRSF10D	63,81	81,21	100,23	109,03	167,85	1,27	1,57	1,71	2,63								
	8795	TNFRSF10B	36,50	58,73	54,58	45,47	44,50	1,61	1,50	1,25	1,22								
	55,504	TNFRSF19	15,97	12,01	15,13	6,37	6,00	−1,33	−1,06	−2,51	−2,66								
	7132	TNFRSF1A	15,99	23,77	18,27	5,92	1,80	1,49	1,14	−2,70	− 8,87								
	7133	TNFRSF1B	2,53	2,93	1,90	4,13	5,01	1,16	−1,33	1,63	1,98								
	27,242	TNFRSF21	7,01	6,49	6,71	2,50	2,30	−1,08	− 1,04	−2,80	− 3,05								
	355	FAS	29,00	32,62	29,42	23,35	27,09	1,12	1,01	−1,24	−1,07								
	8797	TNFRSF10A	0,64	1,78	2,56	6,94	8,00	2,77	3,98	10,81	12,46								
	7186	TRAF2	2,45	1,66	2,63	2,7	2,58	−1,48	1,07	1,1	1,05								2,41
	10,131	TRAP1	14,67	14,53	26,94	57,12	83,3	− 1,01	1,84	3,89	5,68	−1,05	− 1,1	− 1,13	−1,1	1,13	1,17	1,35	1,33
	598	BCL2L1	14,76	15,24	8,1	3,22	1,48	1,03	−1,82	−4,59	− 10								
	23,786	BCL2L13	19,51	25,36	20,22	11,06	11,61	1,3	1,04	−1,76	−1,68	1,24	−1,14	− 1,36	−1,02		1,45	1,47	
	599	BCL2L2	11,5	12,4	11,11	15,34	15,44	1,08	−1,03	1,33	1,34								
	4170	MCL1	96,6	144,5	87,54	60,8	35,69	1,5	−1,1	−1,59	−2,71								
	581	BAX	7,43	12,83	12,83	7,69	8	1,73	1,73	1,04	1,08	1,64	1,89	2,27	1,79	2,17	2	2,18	1,36
	637	BID	15,54	22,96	17,53	32,71	43,46	1,48	1,13	2,1	2,8	1,88	3,13	2,64	2,8	2,91	3,44	2,79	2,35
	572	BAD	1,5	3,53	4,16	8,28	9,81	2,34	2,77	5,51	6,52			−1,35	−1,31	−1,31	−1,45		
	573	BAG1	5,64	6,36	9,26	13,21	19,28	1,13	1,64	2,34	3,42								
	9532	BAG2	10,35	9,8	8,06	7,47	5,18	−1,06	−1,28	−1,39	−2	− 1,09	1,02	− 1,11	1,04	1,04	−1,01	1,12	−1,11
	9531	BAG3	8,16	9,16	7,75	8,44	4,57	1,12	−1,05	1,03	−1,78		1,63	1,77	1,61	3,13	3,03	3,6	
	9530	BAG4	16,92	14,3	17,17	12,1	10,24	−1,18	1,02	−1,4	−1,65								
	9529	BAG5	16,42	15,16	15,56	22,07	8,35	−1,08	−1,06	1,34	−1,97								
	7917	BAG6	10,57	10,4	11,5	7,75	13,6	−1,02	1,09	−1,36	1,29								
	54,205	CYCS	69,59	65,88	90,45	139,01	184,24	−1,06	1,30	2,00	2,65	−1,01	− 1,03	−1,13	− 1,03	3,05	1,61	3,87	1,95
	834	CASP1	19,65	22,47	16,12	6,71	4,58	1,14	−1,22	−2,93	−4,29	2,02		2,99		2,93	3,14		
	835	CASP2	3,5	1,48	4,7	6,01	1,79	−2,37	1,34	1,72	−1,95								
	836	CASP3	50,96	62,1	34,29	48,89	16,35	1,22	−1,49	−1,04	−3,12	1,49		2,92	2,54	1,95	2,69	1,72	1,63
	837	CASP4	80,5	108,96	64,46	30,83	37,74	1,35	−1,25	−2,61	−2,13	1,27	2,23	1,53	1,95	2	1,71		
	839	CASP6	5,45	4,79	8,89	9,08	7,84	−1,14	1,63	1,67	1,44								
	840	CASP7	9,37	12,92	12,6	19,61	9,81	1,38	1,34	2,09	1,05								
	841	CASP8	6,68	5,52	4,3	4,34	3,6	−1,21	−1,55	−1,54	−1,85								
	9994	CASP8AP2	11,31	7,88	16,64	15,96	4,38	−1,43	1,47	1,41	−2,58								
	790	CAD	3,29	3799	6,14	6,08	4,23	1,15	1,86	1,85	1,28	1,2	1,2	1,51	1,62	1,33	2,21	1,59	2,2
	7157	TP53	8,19	7,39	5,89	9,1	15,47	−1,11	−1,39	1,11	1,89								
	7158	TP53BP1	18,87	15,85	23,88	6,55	2,26	−1,19	1,27	−2,88	−8,33	1,16	1,16	1,12	1	1,08	1,11	1,03	−1,02
	7159	TP53BP2	15,22	16,12	16,79	9,5	6,16	1,06	1,1	−1,6	−2,47								
	112,858	TP53RK	12,49	12,92	14,16	4,2	2,89	1,03	1,13	−2,97	−4,33			1,32	1,07	1,5		1,33	
	329	BIRC2	59,44	75,94	39,41	13,31	15,26	1,28	−1,51	−4,46	−3,9								
	330	BIRC3	5,02	13,05	2,88	1,37	0,72	2,6	−1,74	− 3,67	−6,92								
	332	BIRC5	1,39	1,92	1,61	17,94	33,88	1,39	1,16	12,94	24,45								
	57,448	BIRC6	41,06	32,36	37,58	15,78	11,23	−1,27	−1,09	−2,6	−3,66								
	331	XIAP	16,57	15,62	13,33	6,27	6,03	−1,06	− 1,24	−2,64	− 2,75								
MAVS	5688	PSMA7	93,32	110,46	118,29	176,88	272,83	1,18	1,27	1,9	2,92	1,33	1,33	1,36	1,39	1,36	1,19	1,21	1,05
	5094	PCBP2	28,42	30,77	27,23	40,7	63,18	1,08	−1,04	1,43	2,22	1,37	1,37	1,47	1,26	1,34	1,54	1,97	−1,02
	57,506	MAVS	4,35	3,25	4,1	2,22	1,72	−1,34	−1,06	−1,95	−2,53	1,11	1,11	1,24	−1,24	1,76	1,86	2,63	1,15

^a^Biological replicates

As presented above, NFκB and c-Rel binding sites were the most significant in the promoter regions of genes that were transiently up-regulated during the early phase. Correspondingly, expression of several key factors of the NFκB pathway was activated at both the RNA and protein levels during the early phase (Fig. 2a). The transcription of all NFκB family members was detectable, and NFκB1 was the most highly expressed. Except REL, all showed very similar expression profiles. Specifically, they were moderately induced during the early phase, but decreased rapidly and became down-regulated after 24 hpi. Among them, expression of RELA, NFκB1 and NFκB2 was also detected at the protein level. Coupled with the increased RNA level at 6 hpi, these proteins were also up-regulated. Unexpectedly the NFκB2 and RELA protein levels remained constant until the late phase in spite of the reduction in transcription. The members of NFκB inhibitor family (IκB) displayed diverse transcription profiles. NFKBIA (IκBα) and NFKBIZ (IkBζ) were the most highly expressed and showed similar expression profiles, transiently up-regulated at 6 hpi but decreased at 12 hpi and were reduced more than 8-fold at 36 hpi. NFκBIB (IκBβ) showed an opposite expression pattern, low in uninfected cells and at 6 hpi, but increased after 12 hpi and became up-regulated more than 16-fold at 36 hpi. Thus, it appears that NFKBIB replaced NFKBIA to be the most highly expressed IκB in the late phase. None of these gene products was detected at the protein level. The expression changes of the inhibitors of NFκB kinases (IKKs) subunit, IKBKB, and its regulatory subunit IKBKG, as well as IKK-related kinases, IKBKE and TBK1, appeared to be coordinated. They were delayed as compared to the expression of NFκBs and IκBs and significant down-regulation of transcription occurred at 24 or 36 hpi. A surprising finding was that the expression of IKBKΒ and TBK1 was up-regulated at the protein level. The IKBKΒ protein was up-regulated already at 6 hpi and remained stable until the late phase while the up-regulation of the TBK1 protein was significant after 24 hpi. The results thus indicate that the positive regulators of the NFκB pathway are activated at both the RNA and protein levels during the early phase as result of the host immediate response to the infection. Following the progression of the infection, these proteins remained up-regulated until 36 hpi although their transcription was suppressed.

The Janus kinase-signal transducer and activator of transcription (JAK/STAT) signaling is another important pathway regulating the innate immune response. Transcription of all six STATs was unchanged up to 12 hpi, but was then down-regulated after 24 hpi (Table 3). Four STAT proteins (STAT1, STAT2, STAT3 and STAT6) were detected and they were up-regulated during the early phase and remained stable or decreased slightly in the late phase. JAKs are important activators of STAT and catalyze the phosphorylation of the STAT proteins. The three JAK kinases, JAK1, JAK2 and TYK2, displayed different expression profiles. JAK1 was the most highly transcribed and increased only slightly at 6 hpi. Then, it decreased to the basic level and remained constant until the late phase. Transcription of both JAK2 and TYK2 increased at 12 hpi. JAK2 decreased during the late phase while TYK2 remained constant. Only JAK1 protein was detected and it decreased slightly during the early phase, but became up-regulated at 24 hpi. The activity of the STAT proteins is also controlled by several negative regulators, including protein tyrosine phosphatase (PTPN), suppressor of cytokine signaling (SOCS) and protein inhibitor of activated STAT (PIAS). Several PTPNs were detected at both RNA and protein levels with inconsistent expression profiles. However, several Importins and Ran, required for nuclear translocation of STATs, were up-regulated at both at the RNA and protein levels during the infection.

Apoptosis pathways are extensively regulated during Ad2 infection. Our RNA sequencing results showed that transcription of more than 60% of genes that are directly involved in apoptosis were down-regulated, whereas only 20% were up-regulated in the late phase (data not shown). Transcription of most TNF family ligands was undetectable or at a very low level except for TNFSF15 and TNFSF4 (Table 3). Both of them decreased after 12 hpi, although TNFSF15 was transiently induced more than 6-fold at 6 hpi. Numerous TNF receptor superfamily members were expressed at the transcriptional level with diverse expression profiles. TNFRSF11B and TNFRSF12A were the most highly expressed receptors, their RNA levels decreased at 12 hpi and were then more than 25- and 6-fold down-regulated at 36 hpi. Unfortunately, none of the TNF receptor superfamily members was detected at the protein level.

Caspases (CASPs) and the Bcl2 families are key players in apoptosis. At the transcriptional level, CASPs showed different expression profiles. Among them, CASP1, 3 and 4 are most highly expressed with similar expression profiles, slightly increased at 6 hpi and then down-regulated. All of these CASPs were detected at the protein level and were up-regulated, opposite to their RNA expression profile. Expressions of most Bcl2 family members were low at the RNA level except for those listed in the Table 3. Transcriptions of most anti-apoptotic BCLs (BCL2A1, BCL2L1, BCL2L13 and MCL1) were down-regulated after a slight increase at 6 hpi. Among them, only BCL2L13 protein was detected which showed 40% increased expression during the late phase. Among pro-apoptotic genes, transcription of BID, BAD and BAX was up-regulated gradually towards the late phase or remained stable. At the protein level, BID and BAX were up-regulated from the early to the late phase, although the RNA level for BAX decreased in the late phase. BAD protein displayed an expression pattern opposite to its RNA. BAD protein level was more than 30% lower than in mock at 12 and 24 hpi although its RNA was 2- and 5-fold higher than in uninfected control at 12 and 24 hpi, respectively.

Inconsistent expression profiles between RNA and protein for the genes involved in MAVS was shown in our previous study [48]. We show here that expression of MAVS is stable at both RNA and protein levels during the early phase, whereas a difference was seen in the late phase. In addition, we have studied the expression of three MAVS regulatory proteins, PSMA7, PCBP2 and TBK1. The expression profiles of the negative regulators PSMA7 and PCBP2 were similar at both the RNA and protein levels, and increased slowly during infection. The positive regulator, TBK1 showed an opposite profile; its RNA was down-regulated at 36 hpi whereas its protein level increased after 24 hpi. In spite of the up-regulation of MAVS and its positive regulator, expression of the target genes (type I interferon genes) was very low or undetectable, suggesting that this antivirus pathway is inactivated during the late phase.

Furthermore, different expression profiles were also observed for galectins LGALS. LGALS3 and 8 (Gal3 and 8) were the most highly expressed among LGALSs and their RNAs were down-regulated after 24 hpi (for more details see Additional file 1: Table S1). However, their proteins remained constant from early to late phase. Galectins have been shown to be involved in innate immune processes [66]. Colocalization of LGALS3 with incoming Ad5 has been observed and its role in Ad5 transport was suggested [67]. Stable expression of LGALS3 has been reported previously in Ad5-infected cells, while it is down-regulated in Ad3-infected cells [68].

## Discussion

Our transcriptomic analysis showed that the alteration of cellular gene expression correlated with the progression of adenovirus infection and that only specific sets of cellular genes were targeted at the different stages of the infection. The most dramatic changes in transcription profile occurred during the early phase although the most significant increase in the number of differentially expressed genes occurred at 24 hpi as infection proceeded into the late phase. About 80% of up-regulated genes at 6 hpi were only transiently induced and their expression decreased after 12 hpi and became down-regulated after 24 hpi. A significant group of these genes encode cytokines, including CXCL1, IL6, CCL2, CCL20, CXCL3, TNFSF15, IL1B, HGF, IL11, CXCL10, RALA, FGF2, FGF7, GDF15, AREG and HGF involved in cellular immune response and cell growth control. Genes involved in apoptosis included MDM2, TNFAIP2, TNFAIP3, TNFAIP6, TNFAIP8, TNFSF15 and BIRC3. Genes involved in cell cycle control, such as CDKN1A, FYN, PLK2, AHR, and RGCC, were also noteworthy. Induction of cytokines by adenovirus has been shown in in vivo studies with rodent and primate animal systems and in human clinical trials [69]. Up-regulation of cytokine expressions most likely represents the host immediate response to Ad2 infection, triggered by the attachment of virus to membrane receptors, the entry process and intracellular transport, as well as Ad2-encoded small RNAs that are produced before any viral protein is translated [70]. It has been shown that MAPK cascades are the key components of the signaling networks that sense cell exposure to environmental stimulation. Stimulation of the Raf/MAPK signaling pathway will activate NFκB and AP- 1. Although consensus transcription factor binding sites analysis showed that NFκB and c-Rel binding site were most significant for the immune response genes in cluster 1, these transcription factors are only responsible for the activation. The rapid decrease of the first wave of up-regulated genes correlated with the expression of Ad2 E1. It has been shown that the interaction of E1A with the coactivators p300/CBP disrupts the histone acetyltransferase activity of p300/CBP and their associated factor PCAF, leading to decreased transcription from a variety of different genes involved in growth arrest, cell differentiation and immune evasion [4, 5, 8, 9, 11–14]. In addition, small E1A forms a complex with hypophosphorylated pRB1 and p300, and recruits the complex to the gene bodies and represses gene expression [17]. Particularly, many proteins in TGFβ, TNF and interleukin signaling pathways have been shown to be enriched among p300-E1A-pRB complex targets. Thus, suppression of the host early antiviral response is mainly credited to the function of small E1A protein.

Following the expression of E1A at 12 hpi, the number of differentially expressed genes increased, and down-regulated genes became more significant (Cluster 12). In addition, 28 genes in cluster 1 that were up-regulated at 6 hpi were down-regulated. Gene ontology analysis showed that genes up-regulated at 12 hpi (Cluster 6 and 7) were enriched for S phase genes. In agreement with the fact that E2F is a target for E1A-mediated activation, E2F transcription factor binding sites were highly significant among up-regulated genes. Thus, deregulation of the cell cycle is mainly ascribed to the ability of the E1A proteins to bind members of pRB family, permitting E2F to activate genes required in S-phase [3, 7]. Although no significant enrichment of TF binding sites was identified for the suppressed genes at 12 hpi for cluster, the E1A protein undoubtedly plays an important role as discussed above. Many genes in clusters 1 and 12, such as THBS1, CTGF, CYR61, KLF6, KLF10, NFKBIA, ATF3, IL6, and F3 are known to be associated with p300-E1A-pRB [17].

When adenovirus DNA replication reached its efficient mode at 24 hpi, the number of differentially expressed genes was dramatically increased. Significantly, transcription factors CREB and CREBP1:CJUN (ATF2:CJUN) binding sites became abundant in up-regulated genes. E1A has been shown to cooperate with CREB to regulate host cell gene expression [71, 72]. While promotion of ATF2:CJUN-dependent genes expression by the N-terminus of E1A has also been shown [73]. There is a relatively stable period from 24 to 36 hpi as compared to that from 6 to 12 hpi. The consensus Sp1 binding sites are the most common in the up-regulated genes. These results are consistent with our early studies using cDNA microarray [74]. Although no significant consensus transcription factor binding sites were identified for down-regulated genes at 12 hpi, E1A most likely play an important role for the repression of these genes, as discussed above. The most significant transcription factor binding sites for the down-regulated genes at 24 hpi (Cluster 17) were ATF2 and CREB.

Many key regulators of cellular immune response showed different expression profiles at RNA and protein levels. Three important NFκB family members RELA, NFκB1 and NFκB2 were up-regulated at the protein level during the early phase and remained stable until late phase although their transcription was suppressed. However, the fact that the downstream target genes of the NFκB pathway were down-regulated during the late phase indicates that these proteins have lost their functions as transcriptional activators. The dramatic up-regulation of NFKIB may contribute to the inhibition of the NFκB activity. Other post-translational control mechanisms, such as the blocking of the nuclear transport, loss of coactivators such as CBP/P300, p400 and TRAPP due to interaction with the Ad2 E1A protein, may contribute to the block of the NFκB activity [75, 76]. Additional yet unidentified mechanism might also cause the inactivation of the NFκB pathway.

Similar to NFκB pathway, several key players in STAT signaling pathway were also activated at both RNA and protein levels during the early phase of infection. Although their transcription was suppressed during the late phase, their protein levels remained stable. The expression of downstream targets of the STAT pathway differed, however, illustrating the complexity of the regulation of STAT pathway. The activity of STATs has been shown to be modulated by various posttranslational modifications [77, 78]. Upon infection, adenovirus uses several strategies to block the STAT pathway. The viral E1A plays a role in the inactivation of the STAT pathway by binding to STATs, or their coactivator CBP/p300 acetyltransferases [13, 15, 79, 80]. In addition, E1A directly binds to hBre1/RNF20 complex and blocks IFN-induced H2B monoubiquitination of histone 2B, resulting in suppression of ISGs [16]. A recent study shows that C terminus of E1A interacts with RuvBL1/pontin and suppresses RuvBL1/pontin-mediated activation of ISGs [81]. Expression of RuvBL1 at both RNA and protein levels were detected in our study, and they remained stable during infection. Furthermore, the E1B-55 k protein represses expression of IFN-inducible genes which leads to the inhibition of the STAT signaling pathway [82]. E3–14.7 K protein interacts with STAT1 which results in the inhibition of STAT1 phosphorylation and nuclear translocation [83]. Phosphorylated STAT1 has been shown to be sequestered at viral replication centers in the nucleus [84]. The increased expression of STAT proteins indicates that STAT pathway might not be blocked. Furthermore, some ISGs were still actively transcribed even in the late phase, such as ISG15, IFIT1 and IFI6. Their transcriptions increase continued during infection and reached more than 4-fold at 36 hpi as compared to the non-infected control (for more details see Additional file 1: Table S1). Regulation of STAT gene expression apparently reflects the complexity of the battle between the virus and its host.

Although most genes that are directly involved in apoptosis were down-regulated at the transcriptional level during the late phase, several important pro-apoptotic players remained up-regulated at the protein level (CASP3, BAX and BID). The fact that apoptosis is efficiently inhibited during an adenovirus infection indicates that the functions of these proteins must be blocked. To counteract the host defensive apoptotic pathways, adenoviruses have established very efficient mechanisms by encoding their own anti-apoptotic proteins in the E1B and E3 regions [85, 86]. In addition, E1A can also block p53 transcriptional activation through sequestration of p300/CBP [87]. Thus, the regulation of apoptosis is very multifaceted.

The facts that most of downstream genes of immune pathways are down-regulated at RNA level during the late phase even though their key regulators are stable or up-regulated at the protein level, indicate that adenovirus-mediated post-translational mechanisms play an important role. As discussed above, inhibition of STAT pathway represents a good example of how adenovirus has evolved redundant strategies to counteract cellular immune response. By regulating protein modification, blocking of protein-protein interactions, inhibiting of the protein transport to its destination, adenovirus apparently controls host cell antiviral pathways. Last but not least, non-coding RNAs (ncRNAs) are known to be important regulators of various biological processes. Alterations of cellular miRNA and lncRNA expression during Ad2 infection have been studied using RNA-seq [46, 47]. Significant changes in their expression take place after 24 hpi. The strong correlation of ncRNA expression changes with infection progression indicates that ncRNA play important roles. A majority of differentially expressed miRNAs were down-regulated during the late phase. One major mechanism by which miRNA regulates gene expression is by suppression of translation through partial complementarity to 3’ UTRs of mRNA. Thus, down-regulation of miRNAs could lead to altered translation of special sets of proteins. In contrast, most differentially expressed cellular lncRNAs were up-regulated in the late phase while several lncRNAs that are predicted to target immune response genes were down-regulated during the late phase. In addition, a large share of differentially expressed lncRNAs are associated with RNA-binding proteins (RBPs), being involved in posttranscriptional RNA processing and translation regulation. However, how they are regulated, and how they are involved in the regulation of cellular gene expression during adenovirus infection needs to be further addressed.

## Conclusion

There were features common to genes which play important roles in cellular immune pathways. Their expression was stimulated at both RNA and protein levels during the early phase. In the late phase however, their transcription was suppressed while proteins level remained stable. These results indicate that Ad2 and the host use different strategies to regulated cellular immune pathways. A control mechanism at the post-translational level must thus exist which is under the control of Ad2.

## Additional file

Additional file 1: Analysis of differetially expressed cellular genes in Ad2-infected cells.ᅟ(XLS 1117 kb)

## Abbreviations

Ad2: Adenovirus type 2
DAVID: The Database for Annotation, Visualization and Integrated Discovery
FPKM: Fragments per kilobase per million reads mapped
IMR-90 cell: Human lung fibroblast
MS: Mass spectrometry
SILAC: Stable Isotope Labelling of Amino acids in Cell culture
